# Perception of Global Participants of ITEC Nations on Country's Preparedness and Response to COVID-19 Pandemic

**DOI:** 10.3389/fpubh.2022.835330

**Published:** 2022-06-09

**Authors:** Kritika Upadhyay, Sonu Goel, Kathirvel Soundappan

**Affiliations:** ^1^Department of Community Medicine and School of Public Health, Post Graduate Institute of Medical Education and Research (PGIMER), Chandigarh, India; ^2^Faculty of Education and Health Sciences, School of Medicine, University of Limerick, Limerick, Ireland; ^3^Faculty of Human and Health Sciences, Swansea University, Swansea, United Kingdom

**Keywords:** COVID-19, preparedness, response, senior administrators, ITEC

## Abstract

**Background:**

The Coronavirus disease 2019 (COVID-19) pandemic has exposed the public health preparedness and response system across the world. The current study was conducted to gauge the perception of public health professionals of Indian Technical and Economic Cooperation (ITEC) countries regarding the preparedness and responses of their countries in mitigating the COVID-19 pandemic.

**Methodology:**

Three capacity-building programs, namely “Managing COVID-19 Pandemic–Experience and Best practices of India” were conducted by PGIMER, Chandigarh, for public health professionals from ITEC countries from April to May 2021 in which 97 participants from 13 countries have participated. The tools used in the study were adapted from WHO's COVID-19 Strategic Preparedness and Response (SPRP), Monitoring and Evaluation Framework, interim guidelines for Critical preparedness, readiness and response actions for COVID-19, and a strategic framework for emergency preparedness, and finalized using Delphi technique. The overall preparedness of managing COVID-19 was rated using five-point Likert scale, whereas the overall score for the country in combating the COVID-19 pandemic was assessed using 10 point scale.

**Results:**

We found that the perception of public health professionals to government response regarding COVID-19 for fostering improvement on COVID-19 situation was “moderate” with respect to transmission and surveillance mechanism, uniform reporting mechanism, and availability of adequate personal protective equipment (PPE) for health workers. However, the participants rated government response as “poor” in the availability of multisectoral national operational plan, human resource capacity, availability of trained rapid response team (RRT), preparedness in prevention and clinical management, training of healthcare workers, communication and community engagement strategies, facilities to test samples of patients, and transparent governance and administration.

**Conclusion:**

A poor level of preparedness of countries in diverse domains of managing the COVID-19 pandemic was observed. As the global threat of COVID-19 is still looming, great efforts on building a robust preparedness and response system for COVID-19 and similar pandemics are urgently required.

## Introduction

The occurrence of an unknown cause of viral pneumonia in Wuhan, China, in late 2019 has led to the worldwide spread of the disease resulting in a pandemic named Coronavirus disease 2019 (COVID-19). Globally, there have been 239,007,759 confirmed COVID-19 cases, including 4,871,841 deaths as of 14^th^ October 2021 (1). High-income countries reported the maximum number of cases and deaths as compared to the developing and underdeveloped countries (2). The World Bank reported a 5.2% contraction in the global gross domestic product (GDP), the most significant global recession in the last several decades (3).

This unprecedented crisis due to the COVID-19 pandemic has warranted various governments to take extraordinary efforts to combat the transmission and hence reduce the morbidity and mortality associated with the disease (4–6). The developed and developing nations adopted almost similar policies such as restriction of movement across the borders, closure of non-essential businesses, a complete shutdown of offices and institutions and home quarantine, social distancing, and closure of schools and colleges, etc. While the developed countries like the USA (7), UK (8), and Russia (9) had expertise in the management of rising cases, the low and middle-income countries (LMICs) like India (10), Vietnam (11), and Bangladesh (12) faced limitations in resources and expertise to manage the crisis (13). As the cases started rising, the developed countries adopted a pharmaceutical approach, whereas most LMICs used non-pharmaceutical interventions (NPIs) (2).

Even after meticulous measures were taken by the countries, various gaps were identified in the delivery of healthcare services like human resource shortage, increased demand for specialized care, inappropriate personal protective equipment (PPE), overtaxing hospitals and non-healthcare-related issues like loss of employment and financial vulnerability. It has led to inequity between the higher and low socioeconomic households, resulting in the overburdening of the system (14, 15). The COVID-19 pandemic has not just impacted the population's health rather the whole economy. It affected all the sectors of the economy, such as hospitality, tourism and aviation, education, goods manufacturing, supply chain, currency exchange, food and agriculture, healthcare and the pharmaceutical industry, and petroleum and oil industry (16, 17). Hence, it is important for the stakeholders (viz. public health professionals, health authorities, research and medical institute, decision-makers, and healthcare providers) involved in the mitigation of COVID-19 to understand the dynamics of the viral outbreak, which is critical for policy development and practice. Gombos et al. (18) from Hungary found that the formation of research groups helped in translating the scientific findings into relevant information, which is highly significant for the government and policymakers in deciding on various aspects of the mitigation due to the COVID-19 pandemic. Further, Zeliha et al. (19) have stated the role of various stakeholders like local authorities, ministry of health, disease control centers, and research institutions and centers at the national and international level in managing the COVID-19 pandemic along with information dissemination for its preparedness and response. Similarly, centers for diseases control and health protection agencies of various countries are involved in decision making related to redeployment, retention of the staff, testing facility, and PPE, etc., based upon the evidence and expert opinions (20). It has also been found that the COVID-19 challenges policymakers to balance political judgment with the responsible use of expert advice (21). However, the decisions or responses differed across countries. We conducted the current study to assess the perception of public health professionals regarding the preparedness and response of the governments of various Indian Technical and Economic Cooperation (ITEC) countries.

## Materials and Methods

### Study Settings and Duration

A total of three capacity building programs, namely “Managing COVID-19 Pandemic–Experience and Best practices of India” were conducted by the Postgraduate Institute of Medical Education and Research (PGIMER), Chandigarh, an institute of National Importance in India, with technical support from the Ministry of External Affairs, Government of India. These programs were conducted under the flagship International Public Health Management Development Programs (IPHMDP), being conducted since 2016 by the institute. The programs aim at building the capacity of public health professionals of 161 ITEC countries on COVID-19 by illustrating the best practices and experience of the Indian government in managing the COVID-19 pandemic.

### Study Participants

We included all public health professionals, such as academic faculty, mid and senior-level program managers, researchers, and clinicians who were directly or indirectly managing the COVID-19.

### Data Collection Tool and Procedure

We adapted the data collection tool from WHO's COVID-19 Strategic Preparedness and Response (SPRP), Monitoring and Evaluation Framework (22), interim guidelines for Critical preparedness, readiness and response actions for COVID-19 (23), and a strategic framework for emergency preparedness (24), which was later finalized using modified Delphi technique (25). It was administered using an online Google form. The first section of the questionnaire was about the background characteristics of participants, whereas the second part comprised four questions that were intended to evaluate the status of COVID-19 infection in the participant's respective countries. The third section comprised of 15 questions to assess the overall preparedness in terms of managing COVID-19, while the last section had four questions on the overall score for the country in combating the COVID-19 pandemic, challenges by the country in controlling the disease, innovative measures, and suggestions undertaken by countries to control the pandemic. We have developed this questionnaire as a Google form and collected the responses anonymously.

### Data Management and Analysis

The overall preparedness of managing COVID-19 was rated using a five-point Likert scale (completely disagree-1, disagree-2, neither disagree nor agree-3, agree-4, and completely agree-5), whereas the overall score for the country in combating COVID-19 pandemic was assessed using a 10-point scale (1 being the poorest to 10 extraordinary). Further, the overall score is classified as low/mild (<4), moderate (4–7), and excellent (≥7) preparation. In order to evaluate the challenges faced by the country in controlling the pandemic, participants selected the challenges faced in controlling the pandemic.

### Ethics

The Institute Ethics Committee of the PGIMER, Chandigarh, exempted this study from ethical review (IEC-08/2020/1743).

## Results

Table 1 shows the demographic characteristics of participants who opined on their country's response to COVID-19. A total of 97 individuals from 13 countries responded to the survey. The maximum participants who responded to the survey were from Bangladesh, (*n* = 41, 42.3%) followed by Nepal (*n* = 19, 19.6%), and Kenya (*n* = 12, 12.4%). The participants were between the age of 29–44.5 years. Majority were men (*n* = 67, 69.1%), postgraduates (*n* = 55, 56.7%), and specialized in medical sciences (*n* = 77, 79.4%). Majority of the participant's reported their primary role as academic faculty (*n* = 33, 34%) while rests were program managers (*n* = 23, 23.7%), researchers (*n* = 13, 13.4%), patient management (*n* = 12, 12.4%), and students (*n* = 8, 8.2%).

**Table 1:** Demographic characteristics of participants from Indian Technical and Economic Cooperation countries in the study.

		**Number** **(*N* = 97)**	**Percentage** **(%)**
Region/Country	**Asian**
	Bangladesh	41	(42.3)
	Nepal	19	(19.6)
	Bhutan	3	(3.1)
	Afghanistan	1	(1.0)
	Maldives	1	(1.0)
	**African**		
	Kenya	12	(12.4)
	Nigeria	2	(2.1)
	**Eastern Mediterranean**		
	Oman	3	(3.1)
	**South American**		
	Colombia	5	(5.2)
	Mexico	5	(5.2)
	Peru	2	(2.1)
	**Western Pacific**		
	Mongolia	2	(2.1)
	Fiji	1	(1.0)
Median (IQR) age in years		35.0 (29.0, 44.5)	
Gender	Male	67	(69.1)
	Female	30	(30.9)
Highest education	Post graduation	55	(56.7)
	Graduation	42	(43.3)
Median (IQR) years of experience		8.0 (3.0, 15.0)	
Area of specialization	Medical Sciences	77	(79.4)
	Others	22	(20.7)
Primary role in their organization	Academic faculty	33	(34.0)
	Programme manager	23	(23.7)
	Researcher	13	(13.4)
	Patient management	12	(12.4)
	Student	8	(8.2)
	Others	2	(2.1)

*IQR, Interquartile range*.

Table 2 shows the perception of participants on the status of COVID-19 transmission and surveillance mechanisms in their respective countries. In terms of transmission, (*n* = 67, 69.1%) believed it as community transmission and majority (*n* = 68, 70.1%) reported active contact-based surveillance system used in their country during the COVID-19. The majority (*n* = 80, 82.5%) of participants had responded that there is a dedicated website for COVID-19 in their countries.

**Table 2:** Perception of participants about COVID-19 transmission and surveillance mechanism in their country.

**Parameter**		***n*** **(n-97)**	**(%)**
Transmission	Community transmission	67	(69.1)
	Sporadic	19	(19.6)
	Cluster of cases	9	(9.3)
	Don't know	2	(2.1)
COVID-19 surveillance system^a^	Active contact-based	68	(70.1)
	SARI based	37	(38.1)
	Hospital based	35	(36.1)
	Community-based	26	(26.8)
	ILI based	16	(16.5)
	None	5	(5.2)
Presence of dedicated website for COVID-19	Yes	80	(82.5)
	No	12	(12.4)
	Don't know	5	(5.1)

*SARI, Severe acute respiratory illness; ILI, Influenza like illness; ^a^More than one response*.

Table 3 summarizes the perception of participants regarding the COVID-19 response undertaken by their respective countries. Around 37 (38.1%) participants agreed on non-availability or poorly prepared multisectoral national operational plans to mitigate the COVID-19 pandemic in their countries. Most (*n* = 44, 45.3%) reported that resource (equipment and other inventories) and human resource capacity assessment had been poorly assessed to address the COVID-19 pandemic. The adequacy of trained rapid response team (RRT) and their placement at all levels of healthcare were opined by merely 40 (41.2%) participants. Although a structured and uniform format for reporting COVID-19 cases was agreed upon by 60 (61.9%) participants, half (*n* = 50, 51.5%) of the participants responded to the use of epidemiological data on COVID-19 for reviewing the public health interventions and resource allocation at regular intervals. Less than half of the participants (*n* = 45, 46.4%) mentioned the presence of appropriate infection prevention and control strategies and standard operating procedures/guidelines at all entry points in their country. The country's preparedness to reorganize the health systems in prevention and clinical management was mostly inadequate (*n* = 49, 50.5%) as per the participants. With regard to the training of health workers and provision of PPE, the vast majority of participants responded that it was insufficient (*n* = 39, 40.2%) or inadequate (*n* = 59, 60.8 %). Most participants disagreed upon the parameters of risk communication and community engagement strategies adopted, response, and cooperation of the general public to control the COVID-19, adequate testing facility for COVID-19 and delivery of essential healthcare need other than COVID-19. No statistically significant difference was observed in the responses from participants with medical vs. non-medical background except for the implementation of appropriate infection prevention and control strategies (Agree or more: 52 vs. 25%, *p* = 0.04) and excellent response and cooperation from the general public (Disagree or more: 33.8 vs. 75%, *p* = 0.005). Similarly, no significantly different responses were observed based on the primary role of the participant (academic faculty, program manager, researcher, and patient management provider except for the availability of a multisectoral national operational plan (*p* = 0.014) (data not tabulated). The overall median (interquartile) score for measures taken for the control of COVID-19 by the country ranged between 4.5 and 7 (average 6) which did not significantly vary between the above sub-group of participants.

**Table 3:** Perception of participants (*n* = 97) on COVID-19 response undertaken by their countries.

**SN**	**Response**	**Agree or more**		**Neutral**		**Disagree or more**	
		***n***	**(%)**	***n***	**(%)**	***n***	**(%)**
**National/sub-national level coordination, planning and monitoring**							
1.	Availability of multisectoral-national operational plan	23	(23.7)	37	(38.1)	37	(38.1)
2.	Human resource capacity and risk analysis assessment	27	(27.8)	26	(26.8)	44	(45.3)
3.	Transparent governance and administration	40	(41.2)	28	(28.9)	29	(29.9)
**Health systems preparedness**							
4.	Adequate preparedness in reorganization of health systems in prevention and clinical management	28	(28.9)	20	(20.6)	49	(50.5)
5.	Training of healthcare providers	32	(32.9)	26	(26.8)	39	(40.2)
6.	Structured and uniform format for reporting	60	(61.9)	23	(23.7)	14	(14.5)
7.	Adequate facility to test the samples of patients	25	(25.7)	31	(32.0)	41	(42.2)
**Surveillance, rapid response team and case investigation**							
8.	Availability of trained Rapid Response Team at all levels of healthcare	40	(41.2)	27	(27.8)	30	(30.9)
9.	Regular analysis of epidemiological data for reviewing public health interventions	50	(51.5)	30	(30.9)	17	(17.5)
10.	Availability of Standard Operating Procedures at all points of entry for screening and risk communication.	49	(50.5)	30	(30.9)	18	(18.5)
**Infection prevention and control**							
11.	Implementation of appropriate infection prevention and control strategies (like adequate triage system and isolation rooms, trained staffs, and other sufficient materials)	45	(46.4)	25	(25.8)	27	(27.8)
12.	Availability of adequate and appropriate Personal Protective Equipment for healthcare providers	59	(60.8)	18	(18.6)	20	(20.6)
**Risk communication and community engagement**							
13.	Well-developed communication and community engagement strategies.	26	(26.8)	37	(38.1)	34	(35.1)
14.	Excellent response and cooperation of the general public	27	(27.8)	29	(29.9)	41	(42.2)
**Maintaining essential health services**							
15.	Adequate addressal of all essential healthcare needs of the population	29	(30.1)	28	(28.9)	40	(41.1)
	Overall median (interquartile) score for control measures	6.0 (4.5, 7.0)					

Table 4 listed the challenges faced by the countries in control of the COVID-19 pandemic. The majority of the participants mentioned the under testing of susceptible population (*n* = 79, 81.4%), lack of appropriate PPEs (*n* = 69, 71.1%), lack of awareness (*n* = 65, 67%) as the biggest challenges faced in controlling the deadly situation. These were followed by other challenges such as safety and security concerns of healthcare workers, underreporting of cases, the low or poor human resource capacity, poor implementation of public health interventions, poor governance/administration, low inventories other than PPE, and panic due to misinformation.

**Table 4:** Challenges faced during the countries in control of COVID-19 pandemic.

**Parameter**	***n***	**(%)**
Under testing of the susceptible population	79	(81.4)
Lack of appropriate PPEs	69	(71.1)
Lack of awareness in the public	65	(67.0)
Safety and security concerns of healthcare workers	58	(59.8)
Underreporting of cases	52	(53.6)
Poor implementation of public health interventions	51	(52.6)
Poor capacity of human resources	51	(52.6)
Poor multi-sectoral action	48	(49.5)
Poor governance/administration	47	(48.5)
Low inventories other than PPE	44	(45.4)
Panic due to misinformation	31	(32.0)
Others	4	(4.1)
No challenges	2	(2.1)

## Discussion

The COVID-19 pandemic response speckled widely across countries. We set out to measure the perceptions of public health professionals from ITEC countries toward their countries' response and preparedness to mitigate the COVID-19 pandemic. This study is not meant to compare countries with one another. Rather, to help the governments to understand the current status, track and measure the countries' response for developing a policy document for disease prevention and mitigation, which will ultimately strengthen the social, health, and economic sectors affected by COVID-19 and other pandemics. We found that the perception of public health professionals to government response regarding fostering improvement on COVID-19 situation was “moderate,” with respect to transmission and surveillance mechanism, uniform reporting mechanism, and availability of adequate PPE for health workers. However, the participants were rated the government response as “poor” in the availability of multisectoral national operational plan, human resource capacity, availability of trained RRTs, preparedness in prevention and clinical management, communication and community engagement strategies, facilities to test samples of the patient, and transparent governance and administration. Jeffrey et al. (26) also developed a 10-item instrument to help policymakers in designing and implementing COVID-19 prevention and treatment strategies; however, it was limited by the number of variables studied and opinions from a limited number of stakeholders from one country. Oleribe et al. (27) reported that interventions adopted by the government regarding the use of PPEs and management of isolation rooms were poor, which is in sharp contradiction to the current study. Further, we captured the information on community transmission of highly virulent viral strains as the dominant mode of spread in their countries, as reported in other studies (28–30).

In the current study, over one-third of participants responded that the governance and administration in their country were transparent and appropriate to control the COVID-19 pandemic. It is in contrast to the study by Jeffrey et al. (26) and Azlan et al. (31) where respondents trusted their government for the measures taken to control the COVID-19 pandemic and the health systems in optimum utilization of medical resources. Globally, the public perception of government decisions in the form of reports and statistics, or other approaches has scored the above average, which also contradicts the findings of the current study (26).

The multisectoral national operational plan to mitigate the COVID-19 pandemic was successfully implemented by various countries (32–34). In contrast, less than one fourth of participants reported a well-developed operational plan in their respective countries in our study. The rapidly growing imbalance between demand and supply during the COVID-19 pandemic led to scarcities of critical goods, thereby posting challenges in maintaining and restocking supplies (35). Similar to our findings, the workforce exacerbated the shortage of N-95 masks, availability of intensive care unit beds and ventilators to patients was reported in the countries like the USA, Italy, and South Korea (36–39). Further, the implementation of non-pharmacological measures like physical distancing, compliance with facemasks, lockdowns, hand washing, self-isolation, and adoption of other behavioral changes was also difficult (26). The published literature for the pandemics also states that the compliance to public health measures differs across countries based on their socio-cultural norms and belief along with the presence of resources (40). For example, Asians fared better by utilizing massive testing campaigns, aggressive lockdown policies and, contact tracing, as equated to countries in Europe and the America (41). Some countries in Latin and North America and Europe delayed imposing any restrictions and faced severe consequences (42). We did not attempt to undertake an analysis of stakeholder perception between countries. Other studies on healthcare workers perspectives to the government response to COVID-19 stated that appropriate infection prevention measures were adopted during the pandemic, like systematic or streamlined supply and use of PPE, and adoption of prevention guidelines (43, 44). We observed that in one-third of cases, trained RRTs consisting of health professionals was present in their country while two-third opined the presence of structured use of reporting system, similar to other studies (45–47). The preparedness for the reorganization of the health system in preventing the magnitude of COVID-19 has helped in containing the outbreak through a rapid increase in bed capacity, adequate equipment and staffing, triaging mechanism, and safe delivery of primary care services which is contrary to the current study which scored below average (48–50). The most significant challenges faced by countries in the current study reported were poor human resource capacity, lack of multisectoral approach and poor governance similar to the other studies (35, 51). The results highlight the importance of adequate preparedness in context to the mitigation of pandemic. The effective preparedness of countries to tackle such a pandemic is important to prevent paralysis of the existing health systems in delivering effective health services.

### Our Study Had the Following Strengths

First, it collated the experiences and perceptions of public health professionals of different countries toward their country's response during the COVID-19 pandemic, which will be useful for framing appropriate policies for pandemic preparedness and response. Second, the study included all the essential aspects of the prevention and control of COVID-19 in the comprehensive tool used for collecting the responses. Our study is not without limitations. The perception of stakeholders might not truly reflect the country's preparedness toward the COVID-19 pandemic as it was self-reported and dependent upon participants' honesty and recall ability. Further, only public health professionals of 13 countries were included in the survey, of which 61.9% were from two countries namely, Bangladesh and Nepal. Hence, the results might not reflect the opinion of all ITEC nations and might limit generalization. There was a lag period between the actual response and seeking the opinion from the participants. Despite these limitations, the present study provides valuable information about the perception of senior stakeholders about their country's response during the COVID-19 pandemic.

## Conclusion

We identified a low to moderate preparedness of various countries in diverse domains of managing the COVID-19 pandemic. As the global threat of COVID-19 is still to end, great efforts on building a robust preparedness and response system for COVID-19 and similar pandemics are urgently required.

## Data Availability Statement

The original contributions presented in the study are included in the article/supplementary material, further inquiries can be directed to the corresponding author.

## Author Contributions

SG contributed to the concept. SG and KU did a literature search and wrote the manuscript. KS contributed to the writing of the manuscript. All authors contributed to the article and approved the submitted version.

## Conflict of Interest

The authors declare that the research was conducted in the absence of any commercial or financial relationships that could be construed as a potential conflict of interest.

## Publisher's Note

All claims expressed in this article are solely those of the authors and do not necessarily represent those of their affiliated organizations, or those of the publisher, the editors and the reviewers. Any product that may be evaluated in this article, or claim that may be made by its manufacturer, is not guaranteed or endorsed by the publisher.
